# The Evaluation of FDG PET/CT Scan Findings in Patients with Organizing Pneumonia Mimicking Lung Cancer

**DOI:** 10.4274/mirt.03016

**Published:** 2015-06-17

**Authors:** Yurdanur Erdoğan, Berna Akıncı Özyürek, Özlem Özmen, Nilgün Yılmaz Demirci, Sezgi Şahin Duyar, Yeliz Dadalı, Funda Demirağ, Jale Karakaya

**Affiliations:** 1 Atatürk Chest Diseases and Thoracic Surgery Research and Education Hospital, Clinic of Pulmonology, Ankara, Turkey; 2 Atatürk Chest Diseases and Thoracic Surgery Research and Education Hospital, Clinic of Nuclear Medicine, Ankara, Turkey; 3 Beypazarı State Hospital, Clinic of Pulmonology, Ankara, Turkey; 4 Atatürk Chest Diseases and Thoracic Surgery Research and Education Hospital, Clinic of Radiology, Ankara, Turkey; 5 Atatürk Chest Diseases and Thoracic Surgery Research and Education Hospital, Clinic of Pathology, Ankara, Turkey; 6 Hacettepe University Faculty of Medicine, Department of Biostatistics, Ankara, Turkey

**Keywords:** Organizing pneumonia, Lung Cancer, positron emission tomography/computed tomography

## Abstract

**Objective:**

Organizing pneumonia (OP) is a rare lung condition that is characterized by the presence of polypoid tissues due to fibroblastic plugs within respiratory bronchioles, alveolar ducts and sacs. The three main radiologic patterns of OP include typical, solitary-focal and infiltrative forms. The maximum standardized uptake value (SUVmax) on positron emission tomography-computed tomography (PET/CT) may be high in benign conditions such as OP as well as malignant diseases. The aim of our study was to investigate PET-CT characteristics of OP in patients mimicking lung cancer.

**Methods:**

The clinical and radiologic characteristics of 50 patients who were referred to our hospital for PET/CT evaluation due to suspicion of lung malignancy, and who were pathologically diagnosed as OP between 2009 and 2013 were retrospectively reviewed.

**Results:**

The mean age of the patients was 58.2 years. Ninety-six percent of patients (48) were male. Radiologic evaluation revealed 27 (54%) focal involvement, 10 (20%) consolidation with air-bronchogram (typical), 1 (2%) infiltrative and 12 (24%) other types of involvement (multiple nodules and cavitary lesions). The mean SUVmax value of the lesions on PET/CT was calculated as 6.5. Mediastinal lymph node involvement (at least one station) was detected in 76% of our study group with a mean SUVmax value of 3.27.

**Conclusion:**

OP may cause false positive results on PET/CT. However, PET/CT results may be used as a guide for invasive procedures that should be performed when there is suspicion of malignancy.

## Abstract (tr)

**Amaç:**

Organize pnömoni (OP), respiratuvar bronşiyoller, alveoler kanallar ve alveollerde fibroblastik tıkaçların oluşturduğu polipoid yapılarla karakterize histopatolojik bir durumdur. Radyolojik olarak OP’nin tipik, soliter-fokal ve infiltratif olmak üzere 3 karakteristik görünümü vardır. Pozitron Emisyon Tomografi-Bilgisayarlı Tomografisi’de (PET-BT) yüksek FDG tutulumu malin hastalıklarda olduğu gibi OP gibi benin hastalıklarda da görülebilir. Bu çalışmada maliniteyi taklit eden OP’li olgulardaki PET-BT bulgularını değerlendirmeyi amaçladık.

**Yöntem:**

Çalışmamızda 2009-2013 yılları arasında Atatürk Göğüs Hastalıkları ve Göğüs Cerrahisi Eğitim Araştırma Hastanesine dış merkezlerden akciğer grafisi veya toraks bilgisayarlı tomografisinde akciğer malinitesinden şüphelenilerek, ileri tetkik ve tedavi amaçlı yönlendirilen ve malinite ön tanısı ile PET-BT çekilen 50 OP’li olgu dahil edildi. Retrospektif olarak radyolojik ve klinik özellikleri kayıt edildi.

**Bulgular:**

Çalışmaya dahil edilen olguların yaş ortalaması 58,2 idi. Yüzde 96’sını (48) erkek hastalar oluşturmaktaydı. Radyolojik olarak PET- BT’de 27 (%54) olguda fokal, 10 (%20) olguda hava bronkogramı içeren konsolidasyon (tipik), 1 (%2) olguda infiltratif ve 12 (%24) olguda da diğer görünümler (multiple nodüller, multiple kaviter lezyonlar) saptandı. PET- BT’de lezyonların maksimum standart tutulum değerlerinin (SUVmax) ortalaması 6,5 olarak hesaplandı. Çalışma grubunun %76’sında en az bir istasyonda mediastinal lenf nodu tutulumu saptandı. Lenf nodlarının ortalama SUVmax değeri 3,27 idi.

**Sonuç:**

Organize pnömoniler PET-BT’de yanlış pozitif sonuçlara yol açabilir. Ancak PET-BT maliniteden şüphe ediliyorsa yapılması gereken invaziv yöntemlere yol gösterici olarak kullanılabilir.

## INTRODUCTION

Organizing Pneumonia (OP) is pathologically defined by the presence of buds of granulation tissue composed of fibroblasts, myofibroblasts, fibrin and collagen exudates (Masson bodies) within the respiratory bronchioles, alveolar ducts and sacs.

The etiologies of OP include infectious pneumonia, connective tissue diseases, inflammatory bowel diseases, solid organ transplantation, drugs, radiotherapy or aspiration (1). It is called Cryptogenic Organizing Pneumonia (COP) if an underlying cause cannot be foundorganizing.

Three main radiologic patterns distinguished in OP are typical, solitary-focal and infiltrative forms. Multiple masses, nodules, cavitary lesions, bronchocentric distribution, inverse halo pattern, band-like opacities, pneumatocele and pleurisy may also be detected in patients with OP (2).

PET/CT, which is especially used for diagnosis, staging, evaluation of response to treatment and distant metastasis in malignant diseases, is a hybrid imaging method that combines anatomic and functional features. It is also used for central nervous system, vascular, inflammatory, and infectious diseases. Additionally, it can guide the site to be sampled, and identify the primary focus if there are multiple lesions.

The aim of this study was to investigate PET-CT findings in each radiologic subtype of organizing pneumonia.

## MATERIALS AND METHODS

The clinical and radiologic characteristics of 50 patients who were referred to our hospital for evaluation with PET/CT due to suspected lung malignancy, and who were pathologically diagnosed as OP between 2009 and 2013 were retrospectively reviewed. These patients were referred to our center because of radiologic abnormalities that were unresponsive to antibiotherapy, and mediastinal lymph node enlargement suspicious for malignancy. Thirty-nine trans-thoracic fine needle biopsies, 8 wedge resections, 2 lobectomies and 1 trans-bronchial biopsy were performed for pathological diagnosis.

Whole body scan by Siemens Biography 6 HI-REZ PET/CT scanner (Siemens Medical Solutions, Knoxville, TN, USA) was performed in patients who had fasted at least 6 hours, and had a blood glucose level below 180 mg/dl. The images of 6-8 bed positions from the base of the skull to high-thigh were obtained an hour after the intravenous bolus injection of fluorodeoxyglucose (FDG) with a dosage ranging from 370 MBq to 555 MBq (10-15 mCi). The patients were positioned with the arms above the head. Some patients were given oral contrast for a better visualization of bowels. A whole-body PET study was followed by enhanced whole-body CT study, and it was used for attenuation correction.

Mediastinal lymph node enlargements detected by thorax CT were classified according to the American Thoracic Society guidelines.

Data analysis was performed by IBM SPSS Statistics 21.0 software package. The normality for continuous variables was checked by using Kolmogorov-Smirnov test. Mann Whitney U test was performed to compare the distribution of two groups for numerical data. Kruskal Wallis test was used for comparison of three groups. When comparing mean SUVmax values of radiologic subtypes, the infiltrative group that consisted of only one patient was omitted. Chi-square test was used to examine the difference between groups for categorical variables. Descriptive statistics were presented as median (min-max) for quantitative data, and as frequency (percentages) for qualitative data. A value of p<0.05 was considered as statistically significant.

## RESULTS

The mean age of the patients was 58.2 (±9.39) years. Ninety-six percent of patients (48) were male. The average tobacco consumption of patients was 31.6 packages/year. The medical history of 20 patients revealed environmental exposure to asbestos, and 16 patients stated biomass exposure.

Twenty-two patients were on regular medication for at least one co-morbid disease (diabetes mellitus, hypertension, chronic obstructive lung disease, rheumatoid arthritis, asthma). Breast cancer (1 patient), colon cancer (1 patient), lung tuberculosis (1 patient) and coronary bypass grafting (3 patients) were other diseases detected in patients’ past medical history.

All patients had at least one symptom (cough 68%, dyspnea 28%, chest pain 44%, fatigue 38%, weight loss 30%, fever 22%, sweating 16%, hemoptysis 12%, arthralgia and myalgia 4%.

The physical examinations of 32 patients (76%) were completely normal. Abnormal physical examination signs included crackles (3 patients), rhoncus (4 patients) on chest auscultation, and hypoxemia (3 patients).

Laboratory tests revealed elevated sedimentation rate in 30 patients (60%), high levels of C-reactive protein in 20 patients (40%), gamma glutamyl transferase in 7 patients (14%), and alkaline phosphatase in one patient. The results of sputum and bronchial aspirate smear and culture for acid-fast bacilli, which were performed in all patients at least once, were all negative. Among 15 patients who were evaluated for connective tissue markers, positive cytoplasmic antineutrophilic antibody (c-ANCA) was detected only in one patient.

All patients underwent fiberoptic bronchoscopy. Anthracotic pigmentation was present in five cases. No endobronchial lesion was observed.

OP was related to infection in 8 patients, to radiotheraphy for breast cancer in 1 patient, to granulomatosis with polyangiitis in 1 patient, to medications in 8 patients (beta-blockers in 4, statins in 2, sulfasalazine in 1 patient, and paroxetine in 1 patient. The remaining 32 patients were accepted as cryptogenic. We could not detect a statistically significant difference between PET-CT findings of cryptogenic and secondary organizing pneumonia patients in terms of mean SUVmax values (p>0.05).

The most common sites of involvement were the left upper, right lower and right upper lobes (30%, 26%, 24%, respectively).

**Figure 1:**
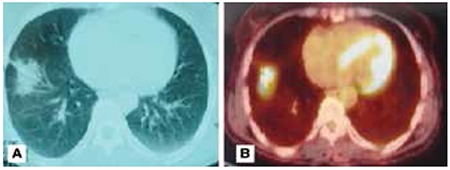
CT and PET-CT scans of a patient with focal lesionA) A focal lesion 3 cm in diameter with irregular borders radiating to the pleural surface in the right lower lobe. B) SUVmax of the lesion on PET/CT was 5.09. Trans-thoracic fine needle biopsy was insufficient for diagnosis, thus, it was surgically removed. The pathology of the lesion was reported as organizing peumonia

**Figure 2:**
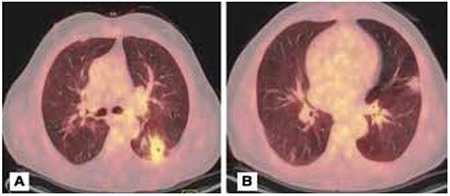
PET/CT scans of a patient with multiple nodules and cavitary lesionA) A cavitary lesion 2.5 cm in diameter in the superior segment of left lower lobe (SUVmax: 7.04). There is low metabolic activity in the left hilar lymph node (SUVmax: 3.38). B) Bilateral multiple nodules, the largest 2.5 cm in diameter (SUVmax: 3.93). The patient underwent trans-thoracic fine needle biopsy, which was reported as organizing pneumonia after pathologic evaluation

**Figure 3:**
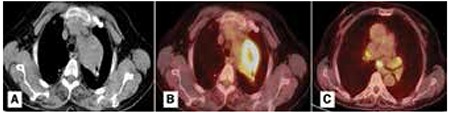
CT and PET-CT scans of a patient with mediastinal massA) A mediastinal lesion of 6.2x4x5 in size, extending to the paramediastinal area from the apical region of the left lung on Thorax CT. B) SUVmax of the lesion on PET/CT was 10.59. The center of the mass was hypo-metabolic. C) High metabolic activity in subcarinal (SUVmax: 6.96), right hilar (SUVmax: 3.32-4.07) and left hilar (SUVmax: 4.12) mediastinal lymph nodes. The pathology of the specimen obtained via trans-thoracic fine needle aspiration biopsy revealed organizing pneumonia

Radiologic findings included focal involvement, consolidation with air-bronchogram (typical), infiltrative, and other types of involvement (multiple nodules and cavitary lesions). Focal lesions consisted of 12 cavitary lesions, 3 ground glass opacities, and 12 solid masses (Figure 1-3). The other radiologic manifestations in our study were multiple cavitary nodules seen in 1 patient, and multiple solid nodules in 11 patients (Figure 2) (Table 1). The mean SUVmax values of radiologic subtypes (typical, focal and the others) were statistically similar (p=0.142) (Table 2). The mean diameter of the primary lesion was 37.7 mm. The mean SUVmax value of the lesions on PET/CT was calculated as 6.5. Mediastinal lymph node involvement (at least one station) was detected in 38 patients (76%). High SUVmax values (SUVmax>2) were observed in N1, N2 and N3 lymph nodes in 37, 30 and 2 patients, respectively. The ratio of N1 and N2 lymph node involvement in different radiologic subtypes did not show a statistically significant difference (Table 3). The mean SUVmax value of these lymph nodes was 3.27.

## DISCUSSION

There have been multiple reports of benign thoracic conditions demonstrating hyper-metabolism on F18-FDG PET including granulomatous infections, benign tumors and autoimmune diseases (A). Organizing pneumonia is an inflammatory disease that can mimic lung cancer by causing a positive F18-FDG PET (3,4,5). Our study indicates that secondary and cryptogenic organizing pneumonia should be considered as part of differential diagnosis in patients with false positive results on F18-FDG PET.

The symptoms, clinical presentation, physical examination and radiology findings may also be identical to lung cancer. However, patients with OP are usually younger (aged between 50-60) than those with lung cancer (6,7). Likewise, in our study, the mean patient age was 58.2 (±9.39). Women are influenced as much as men and smoking history is uncommon in disease etiology (6,7,8). However, in our study, only two patients were female and 36 patients had a history of smoking, with an average lifetime exposure of 31.6 packages/year. Hemoptysis, which is exceedingly rare, may raise suspicion of malignancy (9). The most common symptoms in our study group were cough, dyspnea, chest pain and malaise. Hemoptysis was present in six patients. Four of them had solitary lesions while the remaining two had consolidations with air bronchogram.

There are no specific laboratory findings for OP. There is a moderate leukocytosis in half of the patients. The erythrocyte sedimentation rate and C reactive protein levels are increased in about 70-80% of patients. Gamma-glutamyl transferase and alkaline phosphatase levels may be high in relapsing cases (10,11). In our study group, laboratory tests revealed elevated sedimentation rate in 60%, and high levels of C-reactive protein in 40%, gamma glutamyl transferase in 14%, and alkaline phosphatase in 2%. There is no specific connective tissue marker for OP, unless there is an underlying connective tissue disease (10). In our study, positive cytoplasmic antineutrophilic antibody (c-ANCA) was detected only in one patient that showed the underlying cause of OP as granulomatosis with polyangiitis.

Chest X-ray and high-resolution computed tomography are primary imaging methods in the diagnosis of COP. However, chest X-ray may be normal in 4-10% of patients. Three main imaging patterns distinguished in COP are typical (classical), solitary-focal and infiltrative forms. The most frequent and typical imaging profile of COP is in the form of multiple patchy alveolar opacities, with a peripheral and bilateral distribution in the lower lobes. These opacities often migrate spontaneously, and are rarely unilateral. Air bronchogram is usually present in consolidated areas. Bronchioloalveolar carcinoma and primary pulmonary lymphoma should be kept in mind as part of differential diagnosis. Additionally, eosinophilic pneumonia, multifocal pneumonia, alveolar hemorrhage, multiple pulmonary infarcts, alveolar sarcoidosis or ANCA-associated vasculitis can all have a similar appearance (12). Lesions in eight out of ten patients with typical pattern were located in the lower lobes, one in the right middle lobe, and one in the left upper lobe. These lesions mimicked lung cancer due to resistance to antibiotherapy, and high SUVmax value (mean SUVmax: 5.47). COP with focal involvement is usually seen as nodules or masses in upper lobes that may be cavitary. In our study, the mean SUVmax value of focal lesions was found as 6.72. The differential diagnosis should include tuberculosis, granulomatosis with polyangiitis, aspergillosis and septic embolism (12). In 27 (54%) patients with focal involvement, the most common location was upper lobes (61.5%). Cavitation and ground glass opacity was observed in 12 (44.4%) and 3 (11.1%) patients with focal lesions, respectively. A few patients presented with diffuse bilateral infiltration associated with interstitial opacities, and small superimposed alveolar opacities. Alveolar and interstitial opacities may be detected in a variety of interstitial disorders, infectious diseases and lymphangitic carcinomatosis. Only one patient presented with infiltrative pattern, which showed multiple nodules with irregular borders. This patient underwent PET/CT scan due to suspicion of metastatic disease, which showed high FDG uptake of the nodules with a SUVmax value of 3.44.

Multiple masses, nodules, cavitary lesions, bronchocentric distribution, inverse halo pattern, band-like opacities, pneumatocele and pleurisy are other radiologic findings in patients with OP (2.13).

In our study, 11 patients (28.2%) presented with unilateral and/or bilateral multiple solid masses, and 1 (2.7%) patient had multiple cavitary nodules. Mean SUVmax value of these lesions was calculated as 7.96. The differential diagnosis of multiple cavitary nodules should include lung cancer (squamous cell carcinoma, bronchioloalveolar carcinoma), Hodgkin lymphoma, metastatic disease, granulomatosis with polyangiitis, septic embolism, rheumatoid nodules, hydatid cyst, and traumatic pneumatoceles (14). Mean SUVmax values in patients with different radiologic subtypes (typical, focal and others) were statistically similar (p=0.142) (Table 2).

Mediastinal lymphadenopathy is uncommon in COP. Souza et al. reported mediastinal lymphadenopathy in 6 out of 16 patients with COP (15). The presence of lymphadenopathy strengthens the probability of malignancy. Mediastinal lymph node involvement (at least one station) was detected in 38 patients (76%) in our study group. The mean SUVmax value of these lymph nodes was 3.27. We could not detect a statistically significant difference between different radiologic patterns in terms of N1-N2 involvement rate or SUVmax values of lymph nodes (p>0.05). Nevertheless, almost all cases with N2 lymph node involvement (29 out of 30 cases) also had N1 lymph node involvement, suggesting a lymphatic pathway for disease progression.

18F-Fluorodeoxyglucose uptake is not specific for malignancy (16). Various degrees of 18F-FDG uptake have been reported in infiltrative lung diseases, including idiopathic pulmonary fibrosis, collagen vascular disease-associated interstitial pneumonia, drug-induced pneumonia and radiation pneumonia. However, data on increased 18F-FDG uptake in organizing pneumonia is limited (17). The mean SUVmax value of the lesions on PET/CT was calculated as 6.5 (min: 1.71-max: 16.74) for our study group. High 18F-FDG uptake may raise suspicion for malignancy, but histopathologic examination is required for diagnosis due to false positive results. High 18F-FDG uptake in organizing pneumonia was previously reported by Tateishi et al. They also proved that the patients with air-space consolidation had a significantly higher maximal SUV than those without consolidation (17). In the study by Tateishi et al., the most common CT finding was airspace consolidation with ground glass attenuation. The lowest median SUVmax value was observed in the group of patients with typical radiologic involvement in our study. However, we could not show a significant difference in median SUVmax values between radiologic subtypes. Our study group included cases mimicking malignancy, therefore, the number of cases with typical radiologic appearance were proportionally lower while the number of focal, multiple cavitary and nodular lesions were higher.

There are studies indicating that enhanced 18F-FDG accumulation and retention index, obtained by dual time point 18F-FDG PET scan, reflect disease activity in COP (16,18). While PET-CT may be helpful in predicting prognosis in OP, histopathological evaluation remains to be absolutely necessary in differential diagnosis, due to dramatic differences in both prognosis and therapy (5,16,18).

## CONCLUSION

All radiologic subtypes of organizing pneumonia may demonstrate hyper-metabolism on 18F-FDG PET scan. Mediastinal lymph nodes may also show high SUVmax values in all subtypes. In this study, SUVmax values were statistically similar in both cryptogenic and secondary organizing pneumonia. Thus, 18F-FDG PET-CT cannot be used as a tool in distinguishing cryptogenic OP from secondary OP, or organizing pneumonia from lung cancer. Benign disorders like organizing pneumonia must be kept in mind in differential diagnosis of PET positive lung diseases. Therefore histopathologic examination is a must for definitive diagnosis for these lesions.

## Figures and Tables

**Table 1:**
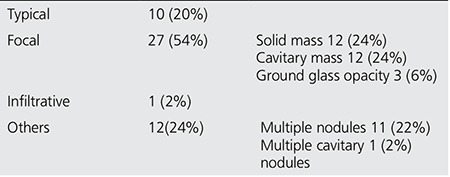
Radiologic characteristics

**Table 2:**
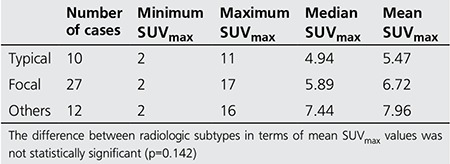
SUVmax values according to radiologic subgroups

**Table 3:**
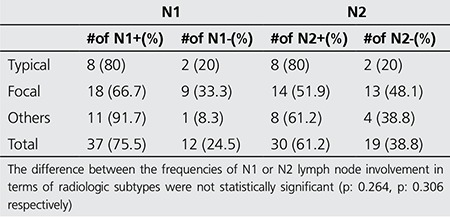
Mediastinal lymph node involvement according to radiologic subtypes
